# NMR Analysis on Molecular Interaction of Lignin with Amino Acid Residues of Carbohydrate-Binding Module from *Trichoderma reesei* Cel7A

**DOI:** 10.1038/s41598-018-38410-9

**Published:** 2019-02-13

**Authors:** Yuki Tokunaga, Takashi Nagata, Takashi Suetomi, Satoshi Oshiro, Keiko Kondo, Masato Katahira, Takashi Watanabe

**Affiliations:** 1 Research Institute for Sustainable Humanosphere (RISH), Kyoto University, Uji, 611-0011 Japan; 2 Institute of Advanced Energy (IAE), Kyoto University, Uji, 611-0011 Japan

## Abstract

Lignocellulosic biomass is anticipated to serve as a platform for green chemicals and fuels. Nonproductive binding of lignin to cellulolytic enzymes should be avoided for conversion of lignocellulose through enzymatic saccharification. Although carbohydrate-binding modules (CBMs) of cellulolytic enzymes strongly bind to lignin, the adsorption mechanism at molecular level is still unclear. Here, we report NMR-based analyses of binding sites on CBM1 of cellobiohydrolase I (Cel7A) from a hyper-cellulase-producing fungus, *Trichoderma reesei*, with cellohexaose and lignins from Japanese cedar (C-MWL) and *Eucalyptus globulus* (E-MWL). A method was established to obtain properly folded *Tr*CBM1. Only *Tr*CBM1 that was expressed in freshly transformed *E*. *coli* had intact conformation. Chemical shift perturbation analyses revealed that *Tr*CBM1 adsorbed cellohexaose in highly specific manner via two subsites, flat plane surface and cleft, which were located on the opposite side of the protein surface. Importantly, MWLs were adsorbed at multiple binding sites, including the subsites, having higher affinity than cellohexaose. G6 and Q7 were involved in lignin binding on the flat plane surface of *Tr*CBM1, while cellohexaose preferentially interacted with N29 and Q34. *Tr*CBM1 used much larger surface area to bind with C-MWL than E-MWL, indicating the mechanisms of adsorption toward hardwood and softwood lignins are different.

## Introduction

Lignocellulosic biomass is the most abundant renewable carbon resource. It consists of structural polysaccharides, cellulose, and hemicelluloses coated with a heterogeneous aromatic polymer, lignin^1^. Recently, the production of bio-based fuels and chemicals from lignocellulosic biomass has attracted increasing attention due to the depletion of fossil resources and environmental issues^2^. To produce biofuels and chemicals by enzymatic saccharification and the fermentation of lignocelluloses, it is necessary to realize pretreatments exposing plant cell wall polysaccharides and subsequent hydrolysis of polysaccharides with a cellulolytic enzyme cocktail simultaneously or prior to fermentation. Highly efficient enzymatic saccharification of lignocellulose with cellulolytic enzymes in a hydrolytic process is a primary key step in achieving lignocellulosic biorefinery process. Typical fungal cellulolytic enzymes, such as cellobiohydrolase and endoglucanase, are composed of catalytic domain (CD) and carbohydrate-binding modules (CBMs) connected with highly glycosylated linker. CBMs play a role in bringing catalytic domains in close proximity to the substrate to improve enzymatic activity^3^. However, CBMs of polysaccharide hydrolases also bind to lignin. The efficiency of enzymatic saccharification, therefore, is strongly decreased^4^. Because the pretreated biomass is usually hydrolyzed by cellulolytic enzymes in the presence of lignin fragments, methods have been extensively explored^5–8^ for protecting enzymes from the unfavorable binding with lignin. The approaches include the addition of masking agents, such as bovine serum albumin^5^, polyethylene glycol^6^, and surfactants^7^, as well as the incorporation of ionic functional groups into lignin^8^. However, no fundamental theories have discussed how to alter the enzyme to avoid the unfavorable binding with lignin because the binding sites of lignin in enzymes are still not understood clearly.

**Figure 1:**
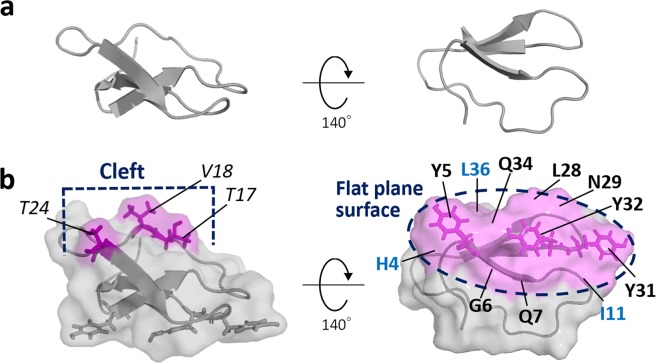
Proposed structure of *Tr*CBM1. (**a**) Cartoon models of *Tr*CBM1 determined by Kraulis *et al*.^19^. Left: a view from the lateral face. Right: a view from the bottom face binding to the cellulose surface. (**b**) Surface models of *Tr*CBM1 looking from the lateral (left) and bottom (right) faces. Left: the cleft, defined as *T17*, *V18*, and *T24* (Italic character, color-coded magenta). Right: the flat plane surface, defined as triplet tyrosine (**Y5**, **Y31**, **Y32**) and **H4**, **G6**, **Q7**, **I11**, **L28**, **N29**, **Q34**, **L36** (Bold character, also color-coded magenta). Carbonyl groups in main chains of **H4** and **I11** are exposed to the same surface as the triplet tyrosine. **L36** is closely located in upper side of **Y5**. The numbering of amino acid residues was based on NMR study for determining the solution structure by Kraulis *et al*. (PDB ID: 2CBH)^19^.

Filamentous fungus *Trichoderma reesei* is known as a hyper producer of cellulolytic enzymes and widely used for commercial-scale production of cellulases and hemicellulases. Up to 60% of totally secreted cellulase is cellobiohydrolase I (*Tr*Cel7A) that bears family 1 CBM as the C-terminal domain (Fig. 1)^9^. Hence, there is a need for detailed understanding of the interaction between *Tr*CBM1 and lignin to solve the nonproductive binding issue and to establish a low-cost, highly efficient enzymatic saccharification process. However, both homologous and heterologous expressions of *Tr*CBM1 as well as its isolation are difficult due to its small molecular size (around 5-kDa). Because of these challenges, there has been no reports on the identification of amino acid residues of *Tr*CBM1 that are involved in binding with lignin without using site-directed mutagenesis that may cause conformational changes of such a small protein. It should be noted that the comparison of intact and *Tr*CBM1-deficient *Tr*Cel7A gives indirect information due to the interference of glycosylated linker^10^.

NMR titration analysis, such as chemical shift perturbation (CSP), is a powerful experimental strategy to identify substrate-binding sites of proteins at amino acid residue resolution^11^. CSP enables comprehensive analysis of interaction sites on the proposed structure of a protein without crucial conformational change. This approach has been used previously for binding site analysis of CBMs with poly- and oligosaccharides, including the interaction site and binding specificity between CBM56 and β-1,3-glucan^12^, CBM32 and chitosan oligosaccharides^13^, as well as CBM6 and xylohexaose^14^.

In this study, we applied CSP to analyze the interaction sites of *Tr*CBM1 against lignins from Japanese cedar and *Eucalyptus globulus*, using^15^N-labeled *Tr*CBM1 prepared as a single protein with correct folding. In addition, interaction of *Tr*CBM1 with cellohexaose was also analyzed by CSP to elucidate differences in the binding mechanisms of *Tr*CBM1 between polysaccharides and lignin. Enhanced understanding these differential interactions will lead to fundamental theory to develop hydrolases having high specificities toward carbohydrates having decreased binding affinity to lignin.

## Results

### Expression and purification of ^15^N-labeled *Tr*CBM1

**Figure 2:**
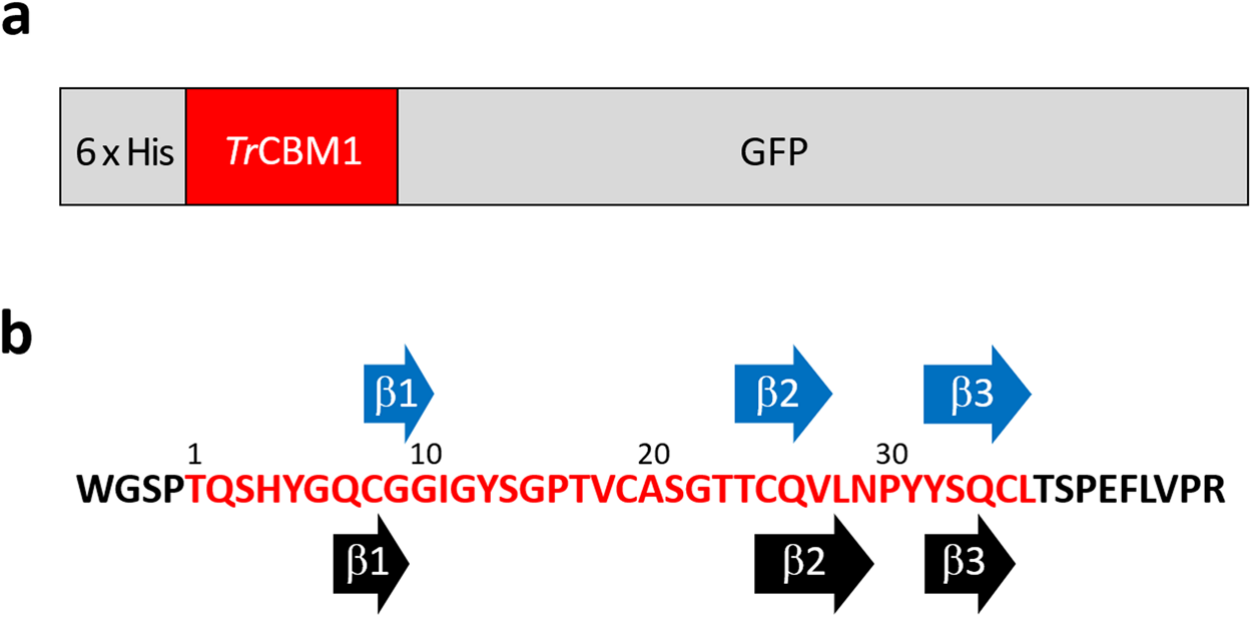
Schematics of His tag-*Tr*CBM1-GFP construct and the primary sequence of *Tr*CBM1 used in this study. (**a**) *Tr*CBM1 was expressed as a fusion protein with His tag and GFP at the N- and C-termini, respectively. (**b**) Amino acid sequences of *Tr*CBM1 (in red) and the residual regions at the N- and C-termini from the enterokinase and thrombin cleavage, respectively (in black). The secondary structures of *Tr*CBM1 predicted by TALOS+ software in this study (blue arrows) and that proposed by Kraulis *et al*.^19^ (black arrows) are also indicated.

**Figure 3:**
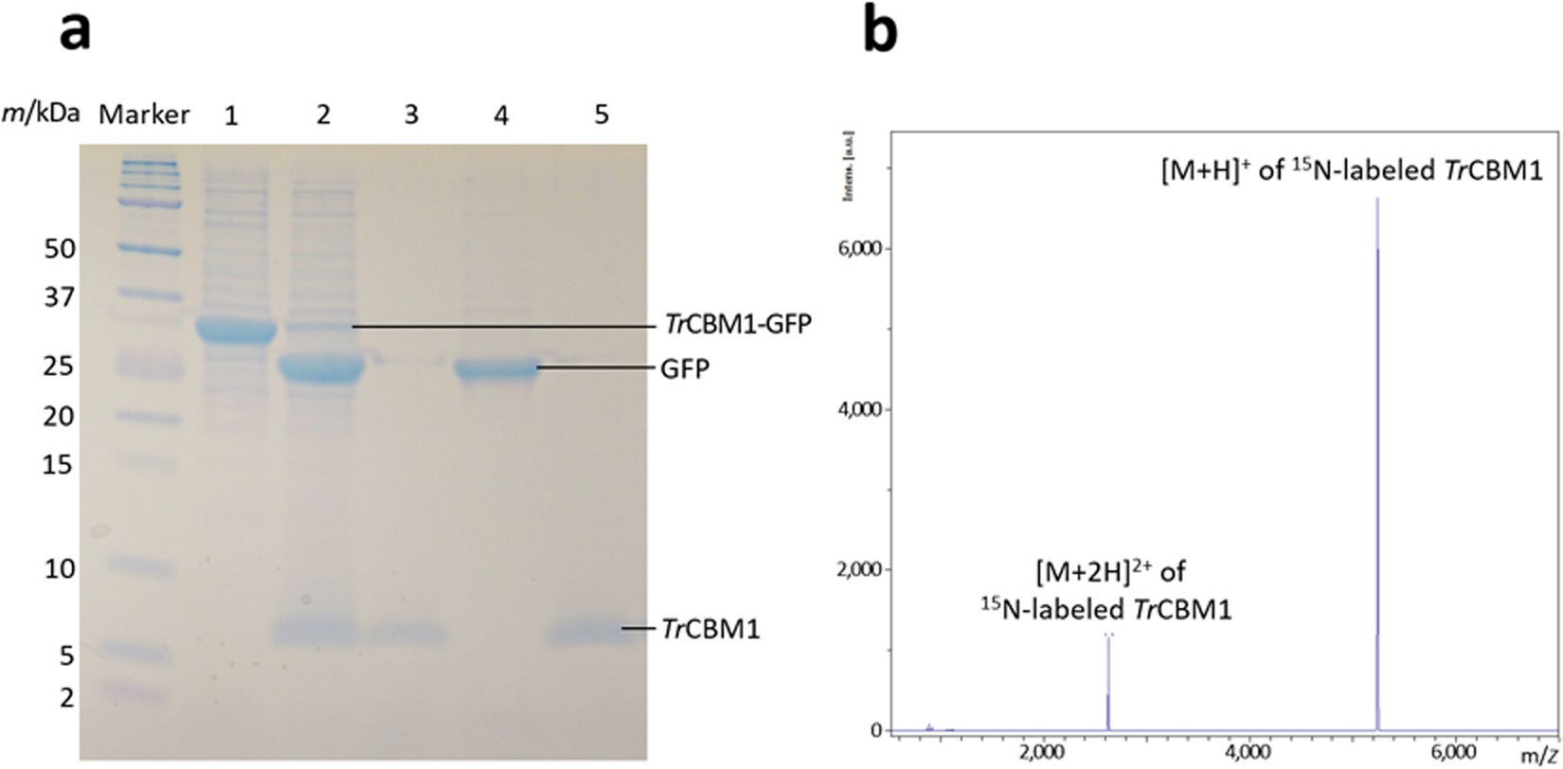
Purity analyses of ^15^N-labeled *Tr*CBM1. (**a**) SDS-PAGE of the ^15^N-labeled target proteins obtained in each purification step. Lanes 1 and 2: the protein fractions before and after cleavage of GFP using thrombin. Lanes 3 and 4: the protein fractions that passed through and were trapped in a benzamidine column, respectively. Lane 5: the *Tr*CBM1 obtained after the final cation exchange chromatography. (**b**) MALDI-TOF-MS spectrum of the purified ^15^N-labeled *Tr*CBM1. The corresponding full-length gel is shown in Supplementary Fig. S4.

^15^N-labeled His tag-*Tr*CBM1-GFP fusion protein was expressed using *Escherichia coli* BL21(DE3) (Fig. 2a). *Tr*CBM1 was cleaved off from His tag and GFP by proteolytic cleavage using enterokinase and thrombin, respectively. Finally, *Tr*CBM1 was purified to a single protein as demonstrated in SDS-PAGE (Fig. 3a) and MALDI-TOF-MS (Fig. 3b). The MALDI-TOF-MS spectrum gave the evidence that the obtained ^15^N-labeled *Tr*CBM1 possessed the correct molecular mass of 5255 expected for ^15^N incorporated protein (Fig. 2b).

**Figure 4:**
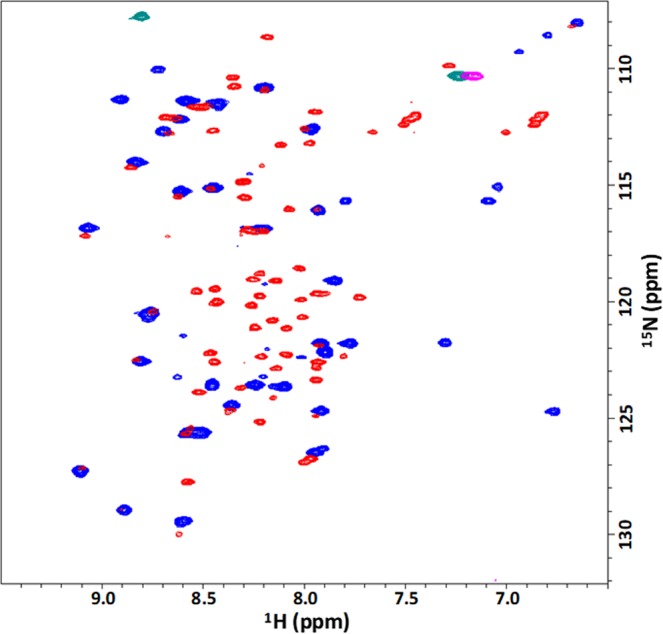
Overlay of 2D ^1^H-^15^N SOFAST-HMQC spectra. Comparison of 100 μM correctly folded *Tr*CBM1 (blue) and 20 μM unfolded *Tr*CBM1 (red) which were expressed using older competent cell stocked for more than five months.

The structures of ^15^N-labeled *Tr*CBM1 were assessed by observing the signal patterns of 2D ^1^H-^15^N SOFAST-HMQC spectra^15^. The ^15^N-labeled *Tr*CBM1 sample that was prepared using *E*. *coli* whose competent cell was stocked for more than five months showed a mixture of 2D ^1^H-^15^N SOFAST-HMQC spectra for both folded and unfolded proteins (Fig. 4). The signals of the folded proteins appeared in the ^1^H-chemical shift range of 6.0–10.0 ppm. The signals of disordered proteins were observed only in the ^1^H-chemical shift range of 8.0–8.5 ppm^16,17^. We conclude that this sample contained folded as well as either partially or fully disordered forms, although the theoretical molecular mass for ^15^N-labeled *Tr*CBM1 was exhibited in MALDI-TOF-MS. ^15^N-labeled *Tr*CBM1 prepared using fresh competent cell gave merely the correctly folded protein signals. The correctly folded ^15^N-labeled, ^13^C/^15^N-labeled, and unlabeled *Tr*CBM1 with single molecular weight were used in this study.

### Spectral assignments of ^13^C/^15^N-labeled *Tr*CBM1

**Figure 5:**
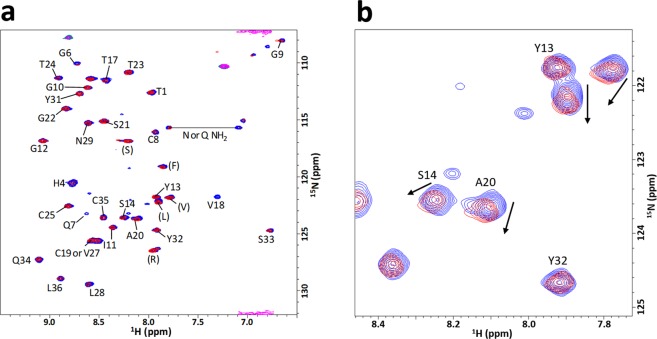
2D ^1^H-^15^N SOFAST-HMQC spectra of NMR titration experiments using ^15^N-labeled *Tr*CBM1 and C-MWL. (**a**) Superposition of 2D ^1^H-^15^N SOFAST-HMQC spectra of 100 μM *Tr*CBM1 in the presence (red) and absence (blue) of C-MWL (2695 μM). The main chain resonances are labeled by corresponding residue number and amino acid type. The amino acid types in parentheses correspond to the amino acid residues in the residual regions from protease cleavage. (**b**) The close-up view of the region exhibited fairly large perturbations.

Spectral assignments of ^13^C/^15^N-labeled *Tr*CBM1 were achieved using a standard sequential assignment procedure. The ^1^H-^15^N HSQC spectrum of ^15^N-labeled *Tr*CBM1 is shown in Fig. 5a with signal assignments. Backbone assignments of *Tr*CBM1 were 89% accomplished with the exception of eight residues. Their signals were not observed because of line broadening that is mainly related to their locations in flexible loop regions. The chemical shifts of backbone atoms (^1^H^N^, ^15^N, ^13^C^α^, ^13^C^β^, and ^13^C′) of *Tr*CBM1 are listed in Supplementary Table S1. The amino acid residues that are in the secondary structures were predicted using TALOS + software based on Table S1^18^, (Fig. 2-b). As a result, ^15^N-labeled *Tr*CBM1 was predicted to have three β-strands: β1 (C8 to G10), β2 (T24 to V27), β3 (Y32 to L36). Previously, Kraulis *et al*. determined the three-dimensional solution structure of the unlabeled *Tr*CBM1 peptide (36 amino acid residues) that was chemically synthesized^19^. According to their report, *Tr*CBM1 has an anti-parallel β-sheet that comprised three β-strands: β1 (Q7 to G9), β2 (C25 to N29), and β3 (Y32 to C35). The structures of *Tr*CBM1s prepared herein and by Kraulis *et al*., therefore, are consistent. Accordingly, we used the solution structure of *Tr*CBM1 determined by Kraulis *et al*. to visualize the results of our NMR titration analyses.

### Analysis of interaction sites of *Tr*CBM1 with MWLs and cellohexaose

The interactions of *Tr*CBM1 with lignin and cellohexaose were comparatively analyzed by NMR titration experiments using ^1^H-^15^N SOFAST-HMQC. We used highly purified milled wood lignins (MWLs) from a softwood, Japanese cedar (*Cryptomeria japonica*) (designated as C-MWL), and a hardwood, *Eucalyptus globulus* (E-MWL). Cellohexaose is an oligosaccharide having the minimum chain length recognizable by *Tr*CBM1^20^, which was used as a model compound of cellulose in the CSP analysis. The addition of excess amounts of MWLs resulted in the disappearance of NMR signals of *Tr*CBM1, which hindered the assignments. Hence, the maximum concentrations of MWLs used for analyses were 2695 and 1200 μM for C-MWL and E-MWL, respectively. The signals of *Tr*CBM1 were still found at these concentrations. Incremental titration to the *Tr*CBM1 solution was carried out using different concentrations of C-MWL (1000, 1839, and 2695 μM), E-MWL (300, 900, and 1200 μM), and cellohexaose (700, 2800, and 5600 μM).

**Figure 6:**
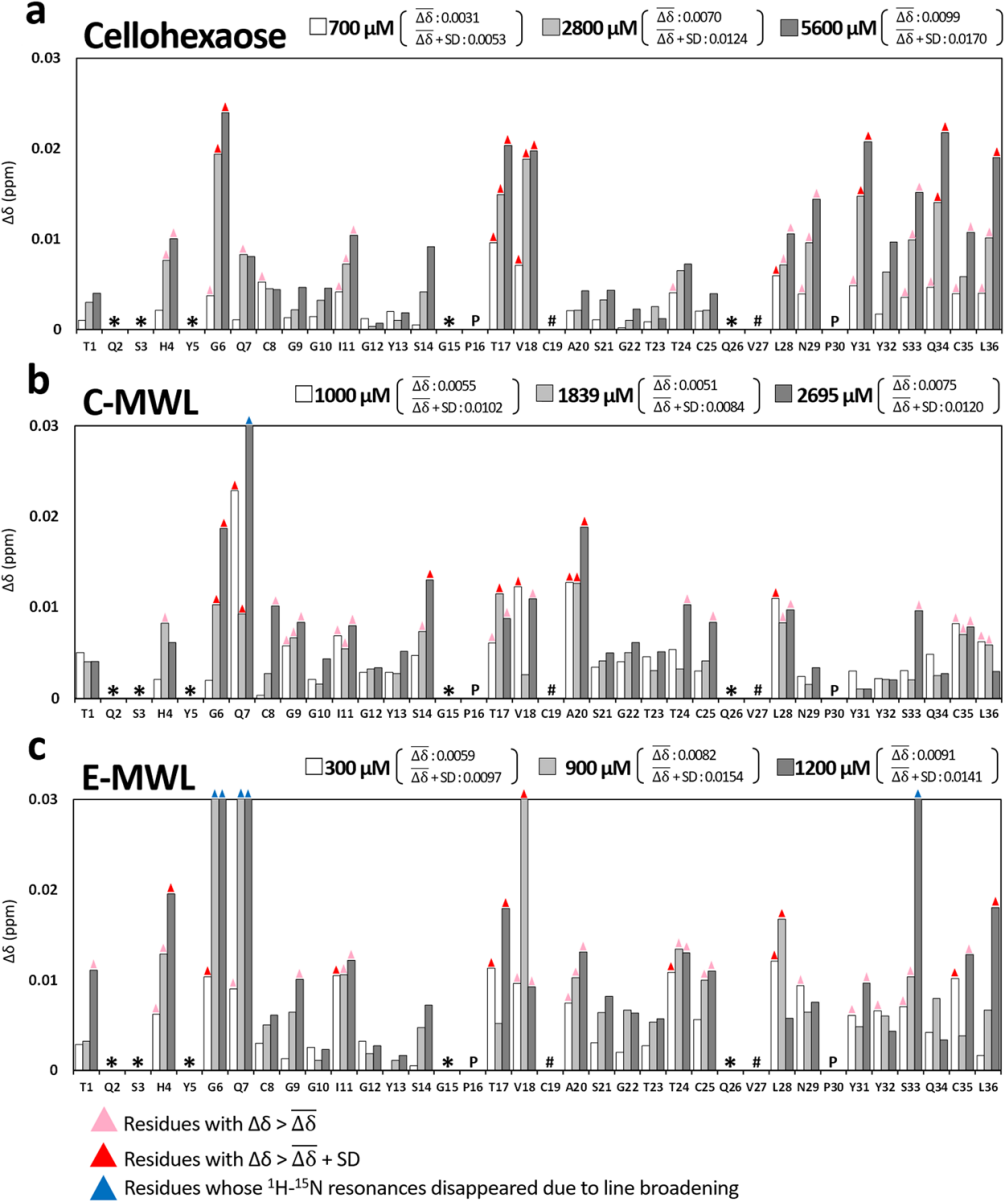
Chemical shift changes (Δδs) of *Tr*CBM1 upon the addition of (**a**) cellohexaose, (**b**) C-MWL, and (**c**) E-MWL. The Δδs were calculated using Eq. (1) in three different concentrations of titrants. The residues that are not assigned are indicated in “*”. Those residues that are overlapped are indicated in “#”. Prolines are indicated in “P”.

^1^H-^15^N SOFAST-HMQC spectra of 100 μM ^15^N-labeled *Tr*CBM1 alone and in the presence of 2695 μM C-MWL were superimposed and are shown in Fig. 5. Upon incremental addition of the titrants to the solution of ^15^N-labeled *Tr*CBM1, several signals clearly exhibited perturbation with the reduction in signal intensity. Further perturbation of the signals was caused by increasing amounts of the titrant. Chemical shift change (Δδ) calculated by the formula (1) is summarized in Fig. 6. The incremental addition of cellohexaose continuously increased Δδ. ^1^H-^15^N SOFAST-HMQC signals of **G6**, *T17*, *V18*, **Y31**, **Q34**, and **L36** exhibited large perturbation without line broadening. In addition, ^1^H-^15^N SOFAST-HMQC signals of **G6**, **Q7**, S14, *T17*, *V18*, A20, and **L28** perturbed greatly upon the addition of C-MWL, while those of **H4**, **G6**, **I11**, *T17*, *V18*, *T24*, **L28**, C35, and **L36** perturbed when E-MWL was added. ^1^H-^15^N SOFAST-HMQC signals of **G6** and S33 were line broadened in the presence of 900 and 1200 μM E-MWL, respectively, whereas the ^1^H-^15^N SOFAST-HMQC signals were not line broadened in the presence of C-MWL. Therefore, distinct binding specificity toward hardwood and softwood lignins was found in the amino acid residues of *Tr*CBM1. When C-MWL and E-MWL were added with concentrations higher than 2695 and 700 μM, respectively, ^1^H-^15^N SOFAST-HMQC signals of **Q7** resulted in line broadening. **Q7**, thus, was involved in direct or indirect interactions with MWLs.

**Figure 7:**
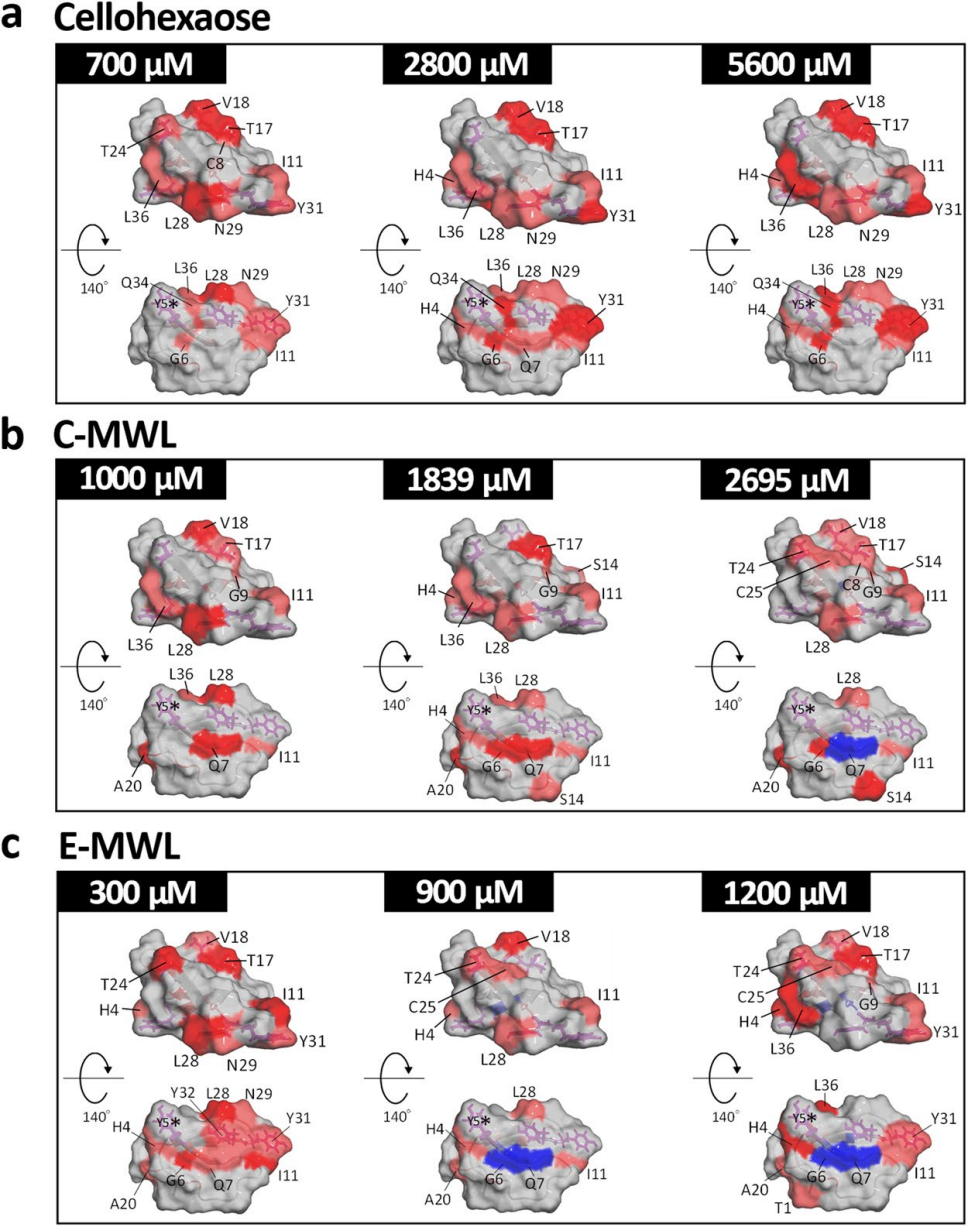
Mapping of cellohexaose and lignin binding sites identified by CSP on *Tr*CBM1. Binding sites of (**a**) cellohexaose, (**b**) C-MWL, and (**c**) E-MWL are shown on the *Tr*CBM1 surface. The residues that exhibited large Δδs are color coded as described in Fig. 6. Two representative views, lateral and bottom faces of *Tr*CBM1, are shown with three concentrations of each titrant. Triplet tyrosine (**Y5**, **Y31**, and **Y32**) and cleft (*T17*, *V18*, and *T24*) are shown using purple stick. The residue **Y5** was not assigned.

The interaction sites of *Tr*CBM1 revealed by NMR titration experiments were mapped on the solution structure of *Tr*CBM1 determined by homonuclear NMR experiments (Fig. 7)^19^. As shown in Fig. 1, *Tr*CBM1 has two major subsites, i.e., the flat plane surface and cleft. Triplet tyrosine (**Y5**, **Y31**, and **Y32**) of *Tr*CBM1 is located on its flat plane surface, which plays a major role in the binding with cellulose. The triplet tyrosine is expected to be a main binding site with lignin due to its hydrophobic nature. However, the perturbations of the ^1^H-^15^N SOFAST-HMQC signals of **Y31** and **Y32** became small, while the assignment of **Y5** was not accomplished. The small Δδ of the triplet tyrosine in the ^1^H-^15^N SOFAST-HMQC spectra is due to the distant location between the aromatic ring in the side chain of tyrosine and ^1^H-^15^N of the main chain, because specific detection of spin coupling of ^1^H-^15^N in peptide bonds was monitored. Although the direct evidence of lignin binding via aromatic ring was not obtained, amino acid residues of the flat plane surface (**H4**, **G6**, **Q7**, **I11**, **L28**, **N29**, **Q34**, and **L36**) exhibited large Δδ upon the addition of MWLs and cellohexaose (Fig. 7). Thus, *Tr*CBM1 interacted with both MWLs and cellohexaose on the flat plane surface. We also found that **G6** and **Q7** were line broadened upon the addition of MWLs, supporting the theory that MWLs strongly bound to *Tr*CBM1 through the flat plane surface. The cleft composed of *T17*, *V18*, and *T24* also interacted with MWLs and cellohexaose. By extensive titration experiments, larger Δδ were consistently observed for *T17* and *V18* than *T24*.

### Binding affinity of *Tr*CBM1 toward cellulose and lignin

Adsorption experiment using Langmuir adsorption model was carried out using MWLs and Avicel. The latter is a commercially available cellulose rich in crystalline regions. A mixture of *Tr*CBM1 with 1%(w/v) of either MWLs or Avicel was incubated at 50 °C for 1 h. The amount of adsorbed *Tr*CBM1 was calculated by subtracting nonadsorbed *Tr*CBM1 from initial loading. The adsorption parameters that were calculated by the formula (2) are summarized in Table 1. The values of *Tr*CBM1 adsorption by Avicel are similar to the previously obtained values using synthesized *Tr*CBM1 analogs^21,22^. Among these titrants, Langmuir affinity constant against *Tr*CBM1 was in the order of E-MWL>C-MWL>Avicel. Therefore, *Tr*CBM1 was found to possess higher affinity toward MWLs than Avicel. The highest Γ_max_ was given by Avicel, indicating that it has a wide surface area and MWLs aggregated in water solution.

**Table 1:** Adsorption parameters of *Tr*CBM1 for C-MWL, E-MWL, and Avicel determined by Langmuir adsorption isotherm.

	Langmuir affinity constant K_L_ (ml/mg)	Amount of adsorption at saturation Γ_max_ (μg/mg)
C-MWL	3.19	54.7
E-MWL	5.93	48.8
Avicel	2.65	63.7

## Discussion

*T*. *reesei* is one of the most important industrial microorganisms for producing cellulolytic enzymes due to its high productivity and high activity for the produced enzymes. Using the cellulolytic enzyme system of *T*. *reesei*, the production of CBHI (Cel7A) reaches up to 60% of the total enzymes^9^. CBHI plays a major role in the catalysis. Its molecular functions including *Tr*CBM1, therefore, have been studied extensively^9^. In cellulose hydrolysis, *Tr*CBM1 plays a crucial role in bringing enzyme close to the substrate, cellulose. However, due to the difficulties of expressing small proteins in *E*. *coli*, the molecular functions of *Tr*CBM1 have been studied using a chemically synthesized analog or as fusion proteins between *Tr*CBM1 and catalytic domain of *T*. *reesei* or other microbes, such as *Talaromyces emersonii* and *Melanocarpus albomyces*^4,23,24^. The exceptions are the studies of Guo and Arslan. They studied the affinities of *Tr*CBM1 to various cellulose substrates^25^ as well as the binding behavior of *Tr*CBM1 to lignocellulosic substrates using an atomic force microscope^26^. These reports described the expression and purification of *Tr*CBM1. However, the molecular mass of the obtained *Tr*CBM1 and whether the obtained *Tr*CBM1 was correctly folded were not presented. These are crucial points, because we found that expression conditions greatly affected the correct folding of *Tr*CBM1. In this study, we focused on the experimental scheme that the binding behavior of *Tr*CBM1 at the molecular level was analyzed using a correctly folded single protein, *Tr*CBM1, as revealed by MALDI-TOF-MS and 2D ^1^H-^15^N SOFAST-HMQC (Figs 3 and 4).

In general, point mutation has been extensively applied for protein-ligand interaction analysis. Indeed, this approach enabled us to identify the key amino acid residues involved in either ligand binding or catalytic activity. In some cases, the substitution of amino acid residues caused undesired changes in the conformation of proteins either partially or entirely. These unwanted structural changes may distort understanding of the actual roles of amino acid residues, especially when the protein of interest is small, such as in the case of *Tr*CBM1^21^. The use of stable isotope labeled proteins in combination with the adapted NMR titration experiments in this study is extraneous from such a disadvantage, giving direct information on the ligand and protein interaction at a molecular level in amino acid resolution.

Our NMR experiments indicated that two subsites of *Tr*CBM1 were the major interaction sites with cellohexaose and MWLs, i.e., the flat plane surface and cleft, (Fig. 7). Previous studies based on site-directed mutagenesis suggested that pyranose rings of cellulose and aromatic rings of lignin bound to *Tr*CBM1 through their triplet tyrosine, i.e., **Y5**, **Y31**, and **Y32**, that are exposed in the flat plane surface by hydrophobic interaction, CH-π, and π–π stacking, respectively, although substitution of the tyrosine residues affected alignment of neighboring amino acid residues^23,24,27^. Our NMR study without the mutagenesis clearly indicated that the amino acid residues around triplet tyrosine (**H4**, **G6**, **Q7**, **I11**, **L28**, **N29**, **Y31**, **Q34**, and **L36**) constituting the flat plane surface exhibited large Δδ. This CSP is explained by changes in shielding effects caused by the interactions of the tyrosine and neighboring amino acid residues with adsorbed cellohexaose or MWLs. Aliphatic OH groups in cellohexaose as well as both aliphatic and phenolic OH groups in MWLs are also the potential binding sites with *Tr*CBM1 through hydrogen bonding and electrostatic interaction^28^. Our NMR study showed that large Δδ was observed in the 2D ^1^H-^15^N SOFAST-HMQC signals of **H4**, **Q7**, and **I11** that are located on the flat plane surface as well as *T17* in the cleft, suggesting that the main chain of **H4** and **I11** as well as the main chain and side chain of **Q7** and *T17* participated in the binding with cellohexaose and MWLs via hydrogen bonding and electrostatic interaction.

Interestingly, a differential binding pattern was observed between cellohexaose and MWLs. **N29** and **Q34** showed large Δδ upon the addition of cellohexaose and thereby were identified as the interaction sites for cellohexaose. This result is consistent with a previous report by Mattinen *et al*.^20^. It was reported that the substitution of **N29** and **Q34** to alanine reduced the affinity toward cellulose over lignin, indicating that **N29** and **Q34** interacted with cellulose more effectively than lignin^23^. In our NMR study, the interactions of **N29** and **Q34** with MWLs were much less remarkable. The hydrophilic side chains of these amino acid residues, therefore, participated in the specific electrostatic interactions and hydrogen bonding with cellulose chains. **G6** and **Q7** in the flat plane surface of *Tr*CBM1 were line broadened upon the addition of MWLs. This phenomenon, however, was not observed for cellohexaose. When MWLs were added to *Tr*CBM1 solution, line broadening was mainly caused by both (1) on- and off-rates of the complex formation and (2) diverse binding states due to the heterogeneity of lignin, which is a good indicator of binding^29^. Additionally, irregular increasing and decreasing of CSP (Fig. 6b,c) support the diverse binding states between lignin and *Tr*CBM1. Therefore, the line broadening of **G6** and **Q7** suggests that the flat plane surface of *Tr*CBM1 played a central role in the binding with lignin.

**Figure 8:**
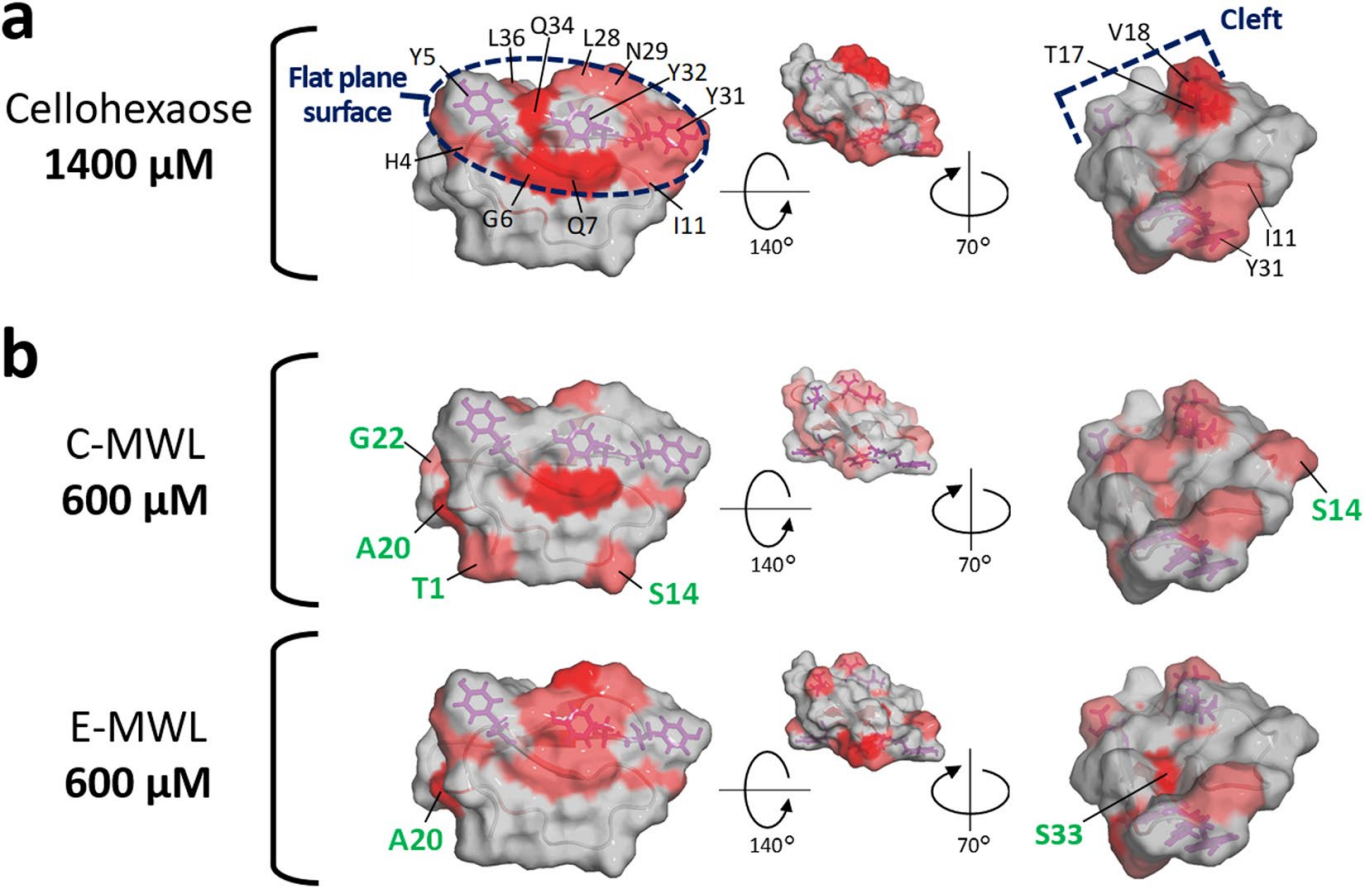
Comparison of interaction property between cellohexaose and MWLs. (**a**) Cellohexaose specifically bound to the flat plane surface and cleft. (**b**) Both MWLs bound to multiple binding sites, some of which are included in the flat plane surface and cleft even in low concentration of titrant. These non-specific binding sites are labeled by green characters.

Cellohexaose bound to the flat plane surface and cleft with high specificity (Fig. 8a). In comparison, MWLs bound to various surface sites, including the flat plane surface and cleft, from much lower concentrations of titrants (Fig. 8b). The cumulative binding of MWLs on multiple exposed sites increased the overall binding affinity to the lignin although the observed CSPs at each site are small (Table 1).

Recently, we found that lignin-binding peptides that can recognize lignin specifically changed their conformation upon the addition of softwood and hardwood lignins to adopt their molecular shapes along with the surface of lignins^30^. Differences in the absorptivity toward softwood and hardwood lignins were also observed for *Tr*CBM1. In addition to the flat plane surface and cleft, C-MWL interacted with the surface of *Tr*CBM1 comprising amino acid residues of T1, S14, A20, and S22. E-MWL interacted with A20 and S33, which are also outside of the flat plane surface and cleft (Fig. 8). Therefore, we conclude that *Tr*CBM1 recognized structural differences of softwood and hardwood lignins having similar weight-average molecular weights (C-MWL: 6254, E-MWL: 5776).

The binding of cellulase to lignin is affected by the structures of exposed surfaces of residual lignin, which results from structural differences of original biomass and pretreatment methods^31,32^. Guo *et al*. compared lignins from six different plants species and concluded that low S/G ratio induced high adsorption capacity, which is consistent with our results (Table 1)^33^. Moreover, high hydrophobicity, phenolic OH groups, and condensed structure of lignin increased adsorption capacity of cellulase, whereas aliphatic OH groups decreased adsorptivity^28,34,35^. Our NMR titration experiments indicated that *Tr*CBM1 bound to lignins through various outer surfaces of the protein, including the flat plane surface and cleft. We found the differences of binding sites between softwood and hardwood lignins. Structural differences caused by pretreatments should also affect the binding behaviors. Thus far, the involvement of triplet tyrosine in lignin binding has been suggested by a combination of point-mutation and adsorption experiments^23,24,27^. These studies suggest the participation of the triplet in lignin binding; however, the role of other protein surfaces in the lignin binding cannot be analyzed and the point mutation may cause conformational changes of the protein. Our CSP study enables comprehensive analysis of interaction sites between the proposed structure of a protein and lignin without crucial conformational change.

Understanding of the flexible molecular recognition mechanism of *Tr*CBM1 bound to polysaccharides and lignins from pretreated biomass softwood and hardwood could contribute to the molecular design of cellulolytic enzymes having controlled affinity to lignin and polysaccharides. The molecular design is indispensable for enzymatic saccharification with the minimum enzyme dosage.

## Conclusion

Nonproductive binding of cellulolytic enzymes to lignin has been a serious issue for enzymatic saccharification of lignocellulosics. Understanding of the adsorption mechanism at the molecular level, however, is still limited. In the present study, we analyzed the interaction sites of correctly folded ^15^N-labeled *Tr*CBM1 with MWLs and cellohexaose through NMR titration experiments. *Tr*CBM1 bound to cellohexaose through the flat plane surface comprising triplet tyrosine as well as cleft with high site specificity. In high contrast, the interaction sites of *Tr*CBM1 with MWLs were spread on the protein surface including the flat plane surface and cleft. Line broadening of **G6** and **Q7** suggests that the flat plane surface of *Tr*CBM1 strongly interacted with MWLs, while hydrophilic amino acid residues, **N29** and **Q34**, interacted with cellohexaose preferentially. The NMR approach using stable isotope labeling could lead to the development of a fundamental theory to design hyper enzymes that preferentially bind to polysaccharides without inactivation by coexisting lignin.

## Materials and Methods

### Materials

*E*. *coli* BL21 (DE3) was purchased from Merck (Darmstadt, Germany). The pRSET-EmGFP vector was obtained from Thermo Fisher Scientific (Waltham, MA, USA). Enterokinase and thrombin were purchased from New England Bio Labs (Ipswich, MA, USA) and GE Healthcare (Chicago, IL, USA), respectively. Cellohexaose was obtained from Toronto Research Chemicals (Toronto, Canada). Other laboratory reagents were purchased from Sigma-Aldrich (St. Louis, MO, USA), Wako Pure Chemical Ltd. (Osaka, Japan), nacalai tesque (Kyoto, Japan), and Cambridge Isotope Laboratories (Tewksbury, MA, USA).

### Preparation of MWLs

Japanese cedar (*Cryptomeria japonica*) and *Eucalyptus globulus* woods were used for the preparation of C-MWL and E-MWL, respectively. The wood meal was extracted using a toluene and ethanol (2:1, v/v) mixture via a Soxhlet extractor at reflux temperature for 10 h. The extracted wood meal was dried at 105 °C for 12 h and finely divided in a vibratory ball mill having constant cooling water under a nitrogen atmosphere for 48 h. The milled wood was extracted using 96% aq. dioxane at room temperature for 24 h. The extract was allowed for solvent evaporation and then freeze dried. The crude MWL was dissolved in 90% aq. acetic acid and then precipitated from distilled water. The precipitates were washed using distilled water and dissolved in a 1,2-dichloroethane and ethanol (2:1, v/v) mixture before they were added to diethyl ether. The precipitates were washed using petroleum ether and allowed for solvent evaporation to give MWL fractions. Molecular weight of MWLs was determined by gel permeation chromatography on three TSK gel supermultipore HZ-M columns (Tosho, Tokyo, Japan) using a Shimadzu instrument equipped with an LC-20AD pump, an SPD M20A diode array detector (Kyoto, Japan). Tetrahydrofuran was used as the eluent at a flow rate of 0.35 ml/min at 40 °C.

### Plasmid construction

The gene of *Tr*CBM1 in Cel7A from *T*. *reesei* and thrombin recognition site was inserted into pRSET-EmGFP vector. The vector map of His tag-*Tr*CBM1-GFP expression plasmid is shown in supplementary (Fig. S1).

### Expression and purification of ^15^N-labeled *Tr*CBM1

*E*. *coli* BL21 (DE3) was transformed by heat shock with His tag-*Tr*CBM1-GFP expression plasmid. The transformant was inoculated to a ^15^N-labeling M9 medium (10 ml), containing ^15^N-NH_4_Cl as the sole nitrogen source and 100 μg/ml ampicillin, before it was precultured at 37 °C for 18 h with shaking at 200 rpm. The culture was used to inoculate ^15^N-labeling M9 medium (750 ml) and further incubated at 37 °C with shaking at 200 rpm, until OD_600_ reached 1.2. Protein expression was induced using 1 mM isopropyl β-1-thiogalactopyranoside (IPTG) at 37 °C for 5 h with shaking at 200 rpm as well.

After centrifugation at 7000 rpm for 15 min (HITACHI, himac CR21GII, R13A rotor), the cells were resuspended by a buffer containing 50 mM sodium phosphate (pH 7.5) and 500 mM NaCl to make a 10% weight per volume solution. This suspension was sonicated, centrifugated at 12000 g for 60 min, and filtered through a 0.45 μm filter. Then, cOmplete His Tag Purification Resin (Roche, Basel, Switzerland) equilibrated with the same buffer was mixed into the solution. The mixture was gently shaken for 15 min on ice and loaded into an open column (Bio Rad, CA, USA). The target protein containing His tag-*Tr*CBM1-GFP was eluted with the same buffer but containing 250 mM imidazole. The fractions containing His tag-*Tr*CBM1-GFP were collected and diluted by tenfold using a 20 mM Tris-HCl buffer (pH 8.0), before they were applied to a 5 ml Hi Trap Q FF column (GE Healthcare, IL, USA) equilibrated with the same buffer. The protein was then eluted from the column using a 0–500 mM NaCl gradient in 20 mM Tris-HCl buffer (pH 8.0) on AKTA prime (GE Healthcare, IL, USA).

Using 10-kDa molecular weight cut off (MWCO) Vivaspin turbo ultrafiltration devices (Sartorius, Göttingen, Germany), the target fraction including His tag-*Tr*CBM1-GFP was concentrated to 1.0 mg/ml in a buffer containing 20 mM Tris-HCl and 50 mM NaCl (pH.8.0). The obtained His tag-*Tr*CBM1-GFP solution was treated by enterokinase (33 U/mg of protein) at 23 °C for 20 h without shaking to cleave the His tag. To remove the cleaved His tag, the reaction mixture was diluted by fivefold using 50 mM sodium phosphate buffer (pH 7.5) containing 500 mM NaCl and incubated with cOmplete His Tag Purification Resin for 15 min on ice. The obtained *Tr*CBM1-GFP solution was then centrifuged at 500 g for 1 min, before the supernatant was filtered through a 0.45 μm filter. Subsequently, the solution was concentrated using 10-kDa MWCO ultrafiltration devices as well to obtain 2.6 mg/ml *Tr*CBM1-GFP dissolved in PBS (-) buffer. The *Tr*CBM1 was separated from GFP by treating with thrombin (50 U/mg of protein) at 22 °C for 20 h without shaking.

The reaction mixture was applied on a 1 ml Hi Trap Benzamidine FF column (GE Healthcare, IL, USA) to remove GFP and thrombin, which were bound to the column. The run-through fraction containing *Tr*CBM1 was collected and buffer exchanged into 20 mM citric acid buffer (pH 3.0) using 3-kDa MWCO ultrafiltration device. The obtained solution was then applied to a 1 ml Hi Trap SP HP column (GE Healthcare, IL, USA) on AKTA prime, before *Tr*CBM1 was eluted using a 0–1 M NaCl gradient in a 20 mM citric acid buffer (pH 3.0). Finally, the obtained *Tr*CBM1 was buffer exchanged into a 100 mM citric acid buffer (pH 5.0) using 3-kDa MWCO ultrafiltration devices.

We used LB medium, a M9 medium containing only ^15^NH_4_Cl (99%, Cambridge Isotope Laboratories), and a M9 medium containing [U-^13^C] glucose (99%, Cambridge Isotope Laboratories)/^15^NH_4_Cl, respectively, to obtain each of the nonlabeled, ^15^N-labeled, and ^13^C/^15^N-labeled *Tr*CBM1s.

The protein concentration was determined by reading the absorbance at 280 nm and using extinction coefficient (11960 M^-1^ cm^-1^), proposed by Pace *et al*.^36^. Molecular mass and purity of the purified ^15^N-labeled *Tr*CBM1 were analyzed by SDS-PAGE and MALDI-TOF-MS using Autoflex III (Bruker Daltonics, MA, USA), respectively. The structures of the purified *Tr*CBM1s were evaluated using 2D ^1^H-^15^N SOFAST-HMQC^15^.

### NMR spectroscopy and spectral assignment of *Tr*CBM1

For NMR experiments, we used the ^13^C/^15^N-labeled *Tr*CBM1 of 150 μM dissolved in 45 mM sodium acetate buffer (pH 5.0), containing 10% D_2_O and 20 μM 2,2-dimethyl-2-silapentane-5-sulfonic acid (DSS). All NMR spectra were recorded at 298 K on a Bruker Avance III 600 spectrometer equipped with a cryogenic probe and Z-gradient (Bruker BioSpin, MA, USA). NMR spectra were processed by NMRPipe/NMRDraw^37^. Spectral analysis was performed by MagRO^38,39^ working with NMRView^40^, following the methods described previously^41^. The assignments of the backbone ^1^HN, ^15^N, ^13^C^α^, ^13^C^β^, and ^13^C′ resonances of *Tr*CBM1 were made using ^1^H-^15^N HSQC, HNCO, HN(CA)CO, HNCACB, CBCA(CO)NH. The secondary structural elements of *Tr*CBM1 were identified using the TALOS+ software^18^.

### NMR chemical shift perturbation analysis

^15^N-labeled *Tr*CBM1 of 100 μM was dissolved in a 100 mM citric acid buffer (pH 5.0), 90% H_2_O/10% D_2_O, and 20 μM DSS. Three different titrants, C-MWL, E-WML, and cellohexaose, were individually titrated into the ^15^N-labeled *Tr*CBM1 solution with incremental concentrations, i.e., C-MWL (600, 1000, 1839, 2695 μM), E-MWL (300, 600, 900, 1200 μM), and cellohexaose (700, 1400, 2800, 5600 μM). To identify the amino acid residues of ^15^N-labeled *Tr*CBM1, which were involved in binding, chemical shift change Δδ (ppm) for each amino acid was calculated using the following equation^42^

1 $$\begin{array}{c}{\rm{\Delta }}{\rm{\delta }}({\rm{p}}{\rm{p}}{\rm{m}})=\sqrt{{(0.17{\rm{\Delta }}{\rm{\delta }}{\rm{N}})}^{2}+{({\rm{\Delta }}{\rm{\delta }}{\rm{N}}{\rm{H}})}^{2}}\end{array}$$

where ΔδN and ΔδNH are chemical shift changes in ^15^N-axis and ^1^H-axis, respectively. Because MWL titrants were dissolved in *d*_6_-DMSO, a control titrant containing *d*_6_-DMSO without MWL was also prepared. The chemical shift changes obtained for MWL titrants were subtracted by those obtained for control titrants to obtain the actual Δδs of *Tr*CBM1 residues for MWLs. The amino acid residues that showed Δδ values larger than the average value ($\overline{{\rm{\Delta }}{\rm{\delta }}}$) were mapped on the proposed *Tr*CBM1 solution structure, which were color coded in pink. The amino acid residues that showed Δδ values larger than the sum of the $\overline{{\rm{\Delta }}{\rm{\delta }}}$ and the standard deviation of Δδ were mapped in red. The amino acid residues whose signals disappeared upon the addition of titrants were mapped in blue. Three-dimensional solution structure of the *Tr*CBM1 was shown using molecular graphics software, PyMOL (Schrödinger, NY, USA).

### Adsorption experiment

Adsorption experiment was employed to evaluate the affinities of *Tr*CBM1 with each of the MWLs and Avicel using Langmuir adsorption isotherm. Sample solutions contained *Tr*CBM1 and one of 1% (w/v) C-MWL, E-MWL, and Avicel in 50 mM citric acid buffer (pH 5.0). The concentration of *Tr*CBM1 was varied as 40, 80, 160, 320, 640, 1280, and 2000 μg/ml with the total volume of 50 μl in a 1.5 ml micro tube. The sample solutions were incubated at 50 °C and were shaken at 1000 rpm for 60 min using thermomixer comfort (Eppendorf, Hamburg, Germany). Subsequently, the sample solutions were centrifuged at 12000 g for 10 min. The free *Tr*CBM1 content in the supernatant was quantified based on the Bradford method using Bio-Rad Protein Assay (Bio-rad, CA, USA). The amount of adsorbed *Tr*CBM1 was calculated by subtracting the amount of free *Tr*CBM1 from that of the initially loaded *Tr*CBM1. C-MWL, E-MWL, or Avicel (1%, w/v) without *Tr*CBM1 were used as a blank. Experiments were carried out at least two times and the results were expressed as average values. Langmuir affinity constant was calculated by the following formula.

2 $$\begin{array}{c}{{\rm{\Gamma }}}_{{\rm{C}}}={{\rm{\Gamma }}}_{{\rm{m}}{\rm{a}}{\rm{x}}}\frac{{{\rm{K}}}_{{\rm{L}}}{\rm{C}}}{1+{{\rm{K}}}_{{\rm{L}}}{\rm{C}}}\end{array}$$

where Γ_C_ is the amount of adsorbed *Tr*CBM1 and Γ_max_ is the amount of adsorbed *Tr*CBM1 at saturation to MWLs and Avicel. K_L_ is the Langmuir affinity constant to MWLs and Avicel. C is the concentration of free *Tr*CBM1 in the supernatant.

## Supplementary information

Supplementary information
