# Supplementary material for: NMR Analysis on Molecular Interaction of Lignin with Amino Acid Residues of Carbohydrate-Binding Module from Trichoderma reesei Cel7A
Source: Sci Rep. 2019 Feb 13;9:1977. doi: 10.1038/s41598-018-38410-9 (PMC6374431; doi:10.1038/s41598-018-38410-9)
Supplement: Supplementary file 1 — Supplementary information [file 41598_2018_38410_MOESM1_ESM.docx]

**Supplementary Materials**

**NMR Analysis on Molecular Interaction of Lignin with Amino Acid Residues of Carbohydrate-Binding Module from *Trichoderma reesei* Cel7A**

**Authors**

Yuki Tokunaga^1^, Takashi Nagata^2^, Takashi Suetomi^1^, Satoshi Oshiro^1^, Keiko Kondo^2^, Masato Katahira^2^ & Takashi Watanabe^1^*

**Affiliations**

^1^Research Institute for Sustainable Humanosphere (RISH), Kyoto University, Uji 611-0011, Japan.

^2^Institute of Advanced Energy (IAE), Kyoto University, Uji 611-0011, Japan.

[*twatanab@rish.kyoto-u.ac.jp](mailto:*twatanab@rish.kyoto-u.ac.jp)

**Plasmid construction**

**Figure S1. Map of the His tag-*Tr*CBM1-GFP expressing plasmid.** DNA sequences of *Tr*CBM1 and thrombin cleavage site were inserted in pRSET-EmGFP vector (Thermo Fisher Scientific).

**Purification process of *Tr*CBM1**

The expressed ^15^N-labeled *Tr*CBM1 comprised a native protein (*Tr*CBM1-A) and a mutant protein (*Tr*CBM1-B) with additional two residues, DR, at the *N*-terminus. The differences in molecular mass ascribed to the additional DR residues were confirmed by MALDI-TOF-MS (Fig. S2). In the final purification step, the two proteins were separated by cation-exchange chromatography (Fig. S3). Throughout this study, we used the native protein (*Tr*CBM1-A) and designated as *Tr*CBM1 in the main text. The differential molecular mass of the native and mutant *Tr*CBM1s were also confirmed by SDS-PAGE (Fig. S4).

**Figure S2. MALDI-TOF-MS spectrum of the purified ^15^N-labeled *Tr*CBM1 containing two fractions.** Differential molecular mass between *Tr*CBM1-A and -B was 277 corresponding to additional ^15^N-labeled residues at the *N*-terminus.

*Tr*CBM1-B

*Tr*CBM1-A

**Figure S3. Cation exchange chromatogram of native and mutant ^15^N-labeled *Tr*CBM1.** Solid line**:** absorbance of 280 nm. Dotted line: Percentage of elution buffer. ^15^N-labeled *Tr*CBM1 mixture in a 20 mM citric acid buffer (pH 3.0) was loaded on a 1ml Hi Trap SP HP column (GE Healthcare, IL, USA). ^15^N-labeled *Tr*CBM1 was eluted using a 0–1 M NaCl gradient in a 20 mM citric acid buffer (pH 3.0). The first eluted fraction was *Tr*CBM1-A, and the second fraction was *Tr*CBM1-B.

**
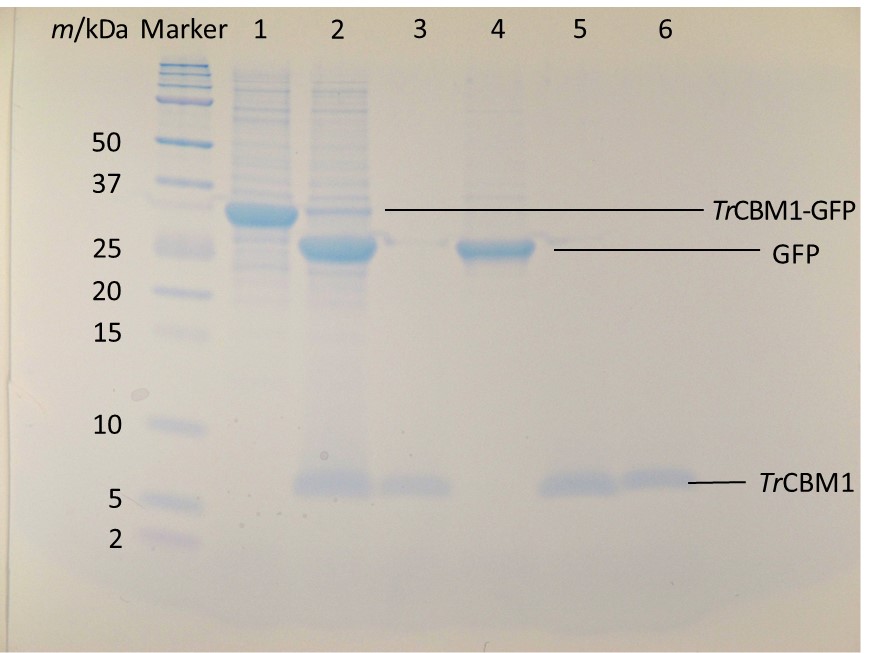
**

**Figure S4. Purity analyses of ^15^N-labeled *Tr*CBM1 by SDS-PAGE; A full-length gel of Figure 3.** Lanes 1 and 2: the protein fractions before and after cleavage of GFP using thrombin. Lanes 3 and 4: the protein fractions that passed through and were trapped in a benzamidine column, respectively. Lanes 5 and 6: *Tr*CBM1-A and B separated by the cation-exchange chromatography on a Hi Trap SP HP column.

**Signal assignment of ^13^C/^15^N-labeled *Tr*CBM1**

**Table S1. Chemical shifts (ppm) for the assigned ^15^N, ^1^H^N^, ^13^Cα, ^13^Cβ, and C’ of *Tr*CBM1**

An asterisk indicates that the chemical shift was not determined.

| Residue | ^15^N | ^1^H^N^ | ^13^Cα | ^13^Cβ | C’ |
| --- | --- | --- | --- | --- | --- |
| (W) | 121.90 | 8.12 | 57.23 | 29.54 | 176.65 |
| (G) | 110.56 | 8.18 | 45.28 | - | 173.69 |
| (S) | 116.88 | 8.11 | 56.76 | 63.42 | 172.95 |
| (P) | - | - | 63.64 | 32.10 | 176.61 |
| T1 | 112.61 | 7.96 | 61.12 | 71.34 | 173.99 |
| Q2 | * | * | * | * | * |
| S3 | * | * | 58.24 | 64.75 | 174.68 |
| H4 | 120.72 | 8.74 | 55.79 | 28.91 | * |
| Y5 | * | * | 61.20 | 36.46 | 175.62 |
| G6 | 110.33 | 8.72 | 44.54 | - | 172.01 |
| Q7 | 123.16 | 8.62 | 55.76 | 27.54 | 174.94 |
| C8 | 116.00 | 7.90 | 55.44 | 46.51 | 171.68 |
| G9 | 108.29 | 6.63 | 44.98 | - | 170.96 |
| G10 | 112.10 | 8.61 | 43.43 | - | 174.60 |
| I11 | 124.62 | 8.32 | 64.60 | 38.32 | 177.83 |
| G12 | 117.23 | 9.07 | 45.31 | - | 173.87 |
| Y13 | 121.93 | 7.90 | 58.38 | 38.76 | 175.93 |
| S14 | 123.87 | 8.29 | 57.42 | 64.09 | * |
| G15 | * | * | * | * | * |
| P16 | - | - | 63.20 | 32.24 | 178.45 |
| T17 | 111.58 | 8.42 | 61.05 | 70.05 | 173.80 |
| V18 | 121.76 | 7.28 | 61.79 | 32.42 | 175.66 |
| C19 | 125.55 | 8.54 | 55.01 | 39.55 | 174.98 |
| A20 | 124.09 | 8.10 | 52.05 | 19.54 | 176.65 |
| S21 | 115.27 | 8.43 | 60.27 | 62.72 | 175.10 |
| G22 | 114.24 | 8.84 | 44.61 | - | 174.05 |
| T23 | 110.92 | 8.16 | 59.35 | 72.34 | 172.62 |
| T24 | 111.56 | 8.87 | 60.31 | 72.08 | 172.78 |
| C25 | 122.45 | 8.80 | 56.09 | 38.69 | 173.94 |
| Q26 | * | * | 54.83 | 30.02 | 175.14 |
| V27 | 125.61 | 8.56 | 63.01 | 32.47 | 176.71 |
| L28 | 129.90 | 8.56 | 56.94 | 42.57 | 176.94 |
| N29 | 115.75 | 8.59 | 53.13 | 37.43 | 173.14 |
| P30 | - | - | 65.86 | 32.02 | 175.93 |
| Y31 | 112.86 | 8.65 | 58.79 | 38.98 | 175.17 |
| Y32 | 124.88 | 7.91 | 61.01 | 42.65 | 172.86 |
| S33 | 124.71 | 6.69 | 56.23 | 65.75 | 171.94 |
| Q34 | 127.49 | 9.06 | 54.01 | 34.69 | 175.03 |
| C35 | 123.64 | 8.42 | 53.76 | 37.61 | 174.73 |
| L36 | 128.91 | 8.87 | 53.64 | 47.50 | * |
| (T) | * | * | * | * | * |
| (S) | * | * | * | * | * |
| (P) | - | - | * | * | * |
| (E) | * | * | 57.05 | 29.95 | 176.41 |
| (F) | 119.63 | 7.85 | 57.57 | 39.57 | 175.27 |
| (L) | 122.97 | 7.92 | 55.31 | 42.35 | 176.62 |
| (V) | 122.40 | 7.76 | 60.46 | 32.54 | 174.20 |
| (P) | - | - | 63.49 | 31.95 | 176.03 |
| (R) | 127.13 | 7.98 | 57.35 | 31.65 | 181.07 |
